# Could technology help us tackle the obesity crisis?

**DOI:** 10.4155/fsoa-2016-0061

**Published:** 2016-10-05

**Authors:** Evan M Forman, Brittney C Evans, Daniel Flack, Stephanie P Goldstein

**Affiliations:** 1 Department of Psychology, Drexel University, Philadelphia, PA, 19104, USA

**Keywords:** application, behavior, intervention, obesity, personalized medicine, smartphone, technology, treatment, weight loss

Technological changes in the last century have brought about profound change in the types and amounts of foods that people consume and the amount of physical activity in which they engage. One result has been a dramatic (and continuing) increase in bodyweight; at present, 2.1 billion people are overweight (BMI ≥25 kg/m^2^) worldwide, and 700 million are clinically obese (BMI ≥30 kg/m^2^). Overweight and obesity are associated with severe health consequences and decreased quality of life, and have been declared an ‘epidemic’ by the WHO [1].

Unfortunately, current intervention approaches have significant shortcomings. Bariatric surgery produces the most weight loss, but has potential for severe medical complications and is often followed by weight regain [2,3]. Weight loss medications are only minimally effective (unless paired with lifestyle interventions) and also come with significant side effects. Dieting on one's own or with the help of a professional (e.g., medical doctor, dietician, nutritionist) produces minimal weight loss and weight regain is the norm [4]. Structured, behavioral weight loss treatments involving nutritional education, behavioral change principles, and psychological strategies are considered the first line of treatment and do produce clinically significant weight loss (5–10%, on average) [5]. However, these treatments are expensive to deliver, require highly trained clinicians (of which there is a shortage), achieve unsatisfactory weight loss for many, and appear to be effective only as long as frequent clinician contact is maintained, after which most participants regain most or all of their lost weight [5]. Of note, the prescriptions that these programs utilize (involving tailored caloric intake goals that are appropriately offset by energy expenditure in the form of physical activity) reliably achieve weight control [6]. The core problem is that most people find it difficult to adhere to these strategies due to biological predispositions to consume high-calorie food and to conserve energy [7]. This difficulty with adherence is multiplied as the rate of weight loss slows or stops and motivation and accountability decrease.

While past technological innovations in food production and labor-saving devices may be driving the obesity epidemic, recent and emerging technological advances also offer promise for addressing the challenges of weight loss. Below are five ways that technology may help us tackle the obesity epidemic.

## Videoconferencing

While continuous in-person meetings with weight control experts produce the best outcomes, in-person meetings are inconvenient and availability of such experts is limited. Videoconferencing allows for meetings to occur virtually, eliminating the need for coaches to be local. Improvements in computing processors, broadband speeds and video compression have made home videoconferencing relatively easy, inexpensive, and high quality [8]. In fact, group videoconferencing is now possible, thus increasing the efficiency of this medium [9]. Thus far, weight control interventions delivered by videoconference have been shown to produce clinically meaningful weight losses [10].

## Exergames

Weight control, especially weight loss maintenance, generally requires a substantial amount of physical activity; a common prescription is one hour of moderate-to-vigorous activity most days of the week. However, many individuals find exercising tedious or unpleasant, and thus are not adherent. ‘Exergames’ are designed to make exercising fun, and use platform sensors (e.g., Wii Fit), a camera, location tracking (global positioning system [GPS] sensor) and/or accelerometry (e.g., a sensor) to track movement. Exergames are appealing because they combine exercise with entertainment, creating social and competitive elements to game play [11]. New developments include virtual reality (technology that creates an immersive and interactive system, providing users with the illusion of entering and/or interacting with a virtual world, for example, Astrojumper, in which virtual, space-themed objects fly toward the player, who must use their own physical movements to avoid collisions) [12], and augmented reality (interface technology using machine vision and 3D graphics to embed virtual content in a real-world view, for example, Pokemon Go, in which players search and catch virtual creatures in their real-world surroundings using a map and a mobile device) [13]. Of note, even games that are not designed to encourage physical activity (i.e., players are drawn by the gaming aspects and not by a desire to be more physically active) can significantly increase physical activity levels. For example, in just 2 months Pokemon Go players walked 4.6 billion km while playing the game.

## Sensor-based tracking

In order to track the calorie intake-expenditure balance necessary for weight loss, exercise and food/drink consumption must be accurately measured. However, traditional methods of tracking these factors are taxing and unreliable, resulting in poor adherence and accuracy. Accelerometers (e.g., in a band, watch or phone) now automatically capture movement and upload data to a tracking application [14]. Automatic tracking of eating may soon be possible through bite, photographic or chemical analysis [15]. For now, web and smartphone apps allow relatively simple tracking using extensive food databases and barcode scanners that are linked to nutritional data [16].

## Mobile & web-based applications

Sophisticated web- and smartphone-based programs offer a suite of features to aid in weight control. Examples include nutritional information, cooking and shopping aides, a library of weight control strategies, prompting and facilitating self-monitoring of activity and eating, personalized goal setting, visualization of behaviors in reference to goals, feedback on behavior, and providing encouragement [17,18]. Some apps take advantage of social reinforcement and accountability by proposing challenges, managing rewards, and encouraging competition among social connections. A recent innovation is the integration of algorithms (mathematical models of behavior that can specify the relationships between multiple variables and continuously adapt based on new data) [19]. These algorithms can be used to achieve sophisticated forms of personalization. For example, algorithm-powered apps can maximize potency by tailoring when an intervention is delivered and what form it will take, and by customizing the physical activity and dietary goals (e.g., based on past performance) [20]. Some experimental systems are also now testing the use of ‘reinforcement learning’ to continuously optimize the choice and nature of interventions based on a particular person's patterns of response to various forms of intervention [21].

## Computerized neurocognitive training

The decisions we make about eating are likely determined much more by ‘implicit’ (quick, automatic) than by ‘explicit’ (slower, reasoned) cognitive processes. As such, ultimately, the most successful interventions may be those that train implicit processes, such as inhibitory control (the mechanism that stops an automatically motivated response), attention, working memory (the mechanism that holds and organizes information), and attitudes. For example, researchers have demonstrated that completing a computerized inhibitory control training (especially over the course of days or weeks) alters eating patterns and perhaps even results in weight loss [22].

## Conclusion & future perspective

New and emerging technologies have promise for confronting the obesity epidemic. Technology offers exciting solutions for promoting weight control behaviors, including apps and devices that make it relatively easy to track physical activity and calorie intake, smartphone apps capable of providing in-the-moment interventions, exergames that make physical activity more motivating and rewarding, personalized weight control coaching via tailored, computerized algorithms, remote obesity interventions capable of delivering effective, low-cost interventions regardless of location, and computerized training programs that improve basic cognitive capacities necessary for exerting behavioral self-control. Yet, there are two important limitations on the excitement we should have about the capability of technology to tackle the obesity epidemic. First, virtually none of the solutions described above has been subjected to adequate empirical scrutiny. For example, Exergames have been shown to increase physical activity, but only in smaller trials that are not well controlled [23]. Web- and mobile-based weight loss programs have proven to be effective, though less so than in-person interventions, and not necessarily for weight loss maintenance [24]. Cognitive training programs appear to shift eating patterns, but it is not yet clear for how long or how profoundly or for whom [25]. Second, certain types of behavioral change may depend on the type of accountability and support that can only be delivered by a person. Perhaps the ultimate answer lies in some combination of technology and human touch, in other words, what some have called ‘touch plus tech’ [26].

## References

[1] (2000). *Obesity: Preventing and Managing The Global Epidemic*.

[2] Herron D, Bloomberg R (2006). Complications of bariatric surgery. *Minerva Chir.*.

[3] Magro DO, Geloneze B, Delfini R, Pareja BC, Callejas F, Pareja JC (2008). Long-term weight regain after gastric bypass: a 5-year prospective study. *Obes. Surg.*.

[4] Franz MJ, Vanwormer JJ, Crain AL (2007). Weight-loss outcomes: a systematic review and meta-analysis of weight-loss clinical trials with a minimum 1-year follow-up. *J. Am. Diet. Assoc.*.

[5] Butryn ML, Webb V, Wadden TA (2011). Behavioral treatment of obesity. *Psychiatr. Clin. North Am.*.

[6] Jeffery RW, Epstein LH, Wilson GT, Drewnowski A, Stunkard AJ, Wing RR (2000). Long-term maintenance of weight loss: current status. *Health Psychol.*.

[7] Lowe MR (2003). Self-regulation of energy intake in the prevention and treatment of obesity: is it feasible?. *Obes. Res.*.

[8] Birden H, Page S (2005). Teaching by videoconference: a commentary on best practice for rural education in health professions. *Rural Remote Health*.

[9] Azar KM, Aurora M, Wang EJ, Muzaffar A, Pressman A, Palaniappan LP (2015). Virtual small groups for weight management: an innovative delivery mechanism for evidence-based lifestyle interventions among obese men. *Transl. Behav. Med.*.

[10] Ahrendt AD, Kattelmann KK, Rector TS, Maddox DA (2014). The effectiveness of telemedicine for weight management in the MOVE! Program. *J. Rural Health*.

[11] Laine TH, Suk HJ (2016). Designing mobile augmented reality exergames. *Games and Culture*.

[12] Finkelstein S, Nickel A, Lipps Z, Barnes T, Wartell Z, Suma EA (2011). Astrojumper: motivating exercise with an immersive virtual reality exergame. *Presence*.

[13] Silver K (2016). Pokemon Go leading to a ‘population-level’ surge in fitness tracker step counts. http://www.fredericksburg.com/townnews/work/pokemon-go-leading-to-a-population-level-surge-in-fitness/article_f7456f9c-ab2d-54b4-a60d-cd16ffa6b0b0.html?mode=jqm.

[14] Lane ND, Miluzzo E, Lu H, Peebles D, Choudhury T, Campbell AT (2010). A survey of mobile phone sensing. *IEEE Commun. Mag.*.

[15] Zhang W, Yu Q, Siddiquie B, Divakaran A, Sawhney H (2015). ‘Snap-n-Eat’ food recognition and nutrition estimation on a smartphone. *J. Diabetes Sci. Technol.*.

[16] Yusof AF, Iahad N (12–14 June 2012). Review on online and mobile weight loss management system for overcoming obesity. *2012 International Conference on Computer & Information Science (ICCIS)*.

[17] Pagoto S, Schneider K, Jojic M, Debiasse M, Mann D (2013). Evidence-based strategies in weight-loss mobile apps. *Am. J. Prev. Med.*.

[18] Neve M, Morgan PJ, Jones P, Collins C (2010). Effectiveness of web-based interventions in achieving weight loss and weight loss maintenance in overweight and obese adults: a systematic review with meta-analysis. *Obes. Rev.*.

[19] Spruijt-Metz D, Hekler E, Saranummi N (2015). Building new computational models to support health behavior change and maintenance: new opportunities in behavioral research. *Transl. Behav. Med.*.

[20] Lin PH, Wang Y, Levine E (2014). A text messaging-assisted randomized lifestyle weight loss clinical trial among overweight adults in Beijing. *Obesity*.

[21] Rabbi M, Aung MH, Zhang M, Choudhury T (9–11 September 2015). MyBehavior: automatic personalized health feedback from user behaviors and preferences using smartphones. *2015 ACM International Joint Conference on Pervasive and Ubiquitous Computing*.

[22] Lawrence NS, O'sullivan J, Parslow D (2015). Training response inhibition to food is associated with weight loss and reduced energy intake. *Appetite*.

[23] Peng W, Lin J-H, Crouse J (2011). Is playing exergames really exercising? A meta-analysis of energy expenditure in active video games. *Cyberpsychol. Behav. Soc. Netw.*.

[24] Hutchesson M, Rollo M, Krukowski R (2015). eHealth interventions for the prevention and treatment of overweight and obesity in adults: a systematic review with meta-analysis. *Obes. Rev.*.

[25] Allom V, Mullan B, Hagger M (2016). Does inhibitory control training improve health behaviour? A meta-analysis. *Health Psychol. Rev.*.

[26] Schiffman LG, Long MM, Sherman E (2015). High tech versus human touch: an exploration of the perceptions of high technology users of the internet. *Global Perspectives in Marketing for the 21st Century*.

